# Postpartum depression in India: a systematic review and meta-analysis

**DOI:** 10.2471/BLT.17.192237

**Published:** 2017-09-05

**Authors:** Ravi Prakash Upadhyay, Ranadip Chowdhury, Aslyeh Salehi, Kaushik Sarkar, Sunil Kumar Singh, Bireshwar Sinha, Aditya Pawar, Aarya Krishnan Rajalakshmi, Amardeep Kumar

**Affiliations:** a Department of Community Medicine, Room 517, 5th floor, College Building, Department of Community Medicine, Vardhman Mahavir Medical College and Safdarjung Hospital, New Delhi 110029, India.; b Independent Researcher, New Delhi, India.; c School of Health and Human Sciences, Southern Cross University, Queensland, Australia; d Directorate of National Vector Borne Disease Control Programme, New Delhi, India.; e Department of Community Medicine, Lady Hardinge Medical College, New Delhi, India.; f Department of Psychiatry, Drexel University College of Medicine, Philadelphia, United States of America.; g Department of Psychiatry, Patna Medical College, Patna, Bihar, India.

## Abstract

**Objective:**

To provide an estimate of the burden of postpartum depression in Indian mothers and investigate some risk factors for the condition.

**Methods:**

We searched PubMed®, Google Scholar and Embase® databases for articles published from year 2000 up to 31 March 2016 on the prevalence of postpartum depression in Indian mothers. The search used subject headings and keywords with no language restrictions. Quality was assessed via the Newcastle–Ottawa quality assessment scale. We performed the meta-analysis using a random effects model. Subgroup analysis and meta-regression was done for heterogeneity and the Egger test was used to assess publication bias.

**Findings:**

Thirty-eight studies involving 20 043 women were analysed. Studies had a high degree of heterogeneity (*I^2^* = 96.8%) and there was evidence of publication bias (Egger bias = 2.58; 95% confidence interval, CI: 0.83–4.33). The overall pooled estimate of the prevalence of postpartum depression was 22% (95% CI: 19–25). The pooled prevalence was 19% (95% CI: 17–22) when excluding 8 studies reporting postpartum depression within 2 weeks of delivery. Small, but non-significant differences in pooled prevalence were found by mother’s age, geographical location and study setting. Reported risk factors for postpartum depression included financial difficulties, presence of domestic violence, past history of psychiatric illness in mother, marital conflict, lack of support from husband and birth of a female baby.

**Conclusion:**

The review shows a high prevalence of postpartum depression in Indian mothers. More resources need to be allocated for capacity-building in maternal mental health care in India.

## Résumé

**Objectif:**

Fournir une estimation de la charge de la dépression post-partum chez les mères indiennes et étudier certains facteurs de risque liés à cette maladie.

**Méthodes:**

Nous avons recherché dans les bases de données PubMed®, Google Scholar et Embase® des articles, publiés entre l'année 2000 et le 31 mars 2016, sur la prévalence de la dépression post-partum chez les mères indiennes. Nous avons articulé nos recherches autour de vedettes-matière et de mots-clés, sans restrictions de langues. La qualité a été évaluée au moyen de l'échelle d’évaluation de la qualité Newcastle-Ottawa. Nous avons réalisé la méta-analyse à l'aide d'un modèle à effets aléatoires. Une analyse par sous-groupes et une méta-régression ont été effectuées à l'égard de l'hétérogénéité et le test Egger a été utilisé pour évaluer le biais de publication.

**Résultats:**

Trente-huit études portant sur 20 043 femmes ont été analysées. Les études présentaient un degré élevé d'hétérogénéité (I2 = 96,8%) et l'existence de biais de publication a été démontrée (bais Egger = 2,58; intervalle de confiance, IC, à 95%: 0,83-4,33). L'estimation combinée globale de la prévalence de la dépression post-partum était de 22% (IC à 95%: 19-25). Après exclusion de 8 études rendant compte de dépressions post-partum dans les 2 semaines suivant l'accouchement, la prévalence combinée a été estimée à 19% (IC à 95%: 17-22). Quelques petites différences négligeables au niveau de la prévalence combinée ont été constatées selon l'âge de la mère, la situation géographique et le cadre de l'étude. Les facteurs de risques associés à la dépression post-partum qui ont été identifiés incluaient des difficultés financières, la présence de violence domestique, des antécédents de maladie psychiatrique chez la mère, des conflits conjugaux, une absence de soutien de la part du mari et la naissance d'une fille.

**Conclusion:**

La revue a révélé une prévalence élevée de la dépression post-partum chez les mères indiennes. Il est nécessaire d'allouer davantage de ressources au renforcement des capacités en ce qui concerne les soins de santé mentale destinés aux mères indiennes.

## Resumen

**Objetivo:**

Ofrecer una estimación de la carga de la depresión posparto en madres indias e investigar algunos factores de riesgo de la enfermedad.

**Métodos:**

Se realizaron búsquedas en las bases de datos de PubMed®, Google Scholar y Embase® para encontrar artículos publicados desde el año 2000 hasta el 31 de marzo de 2016 sobre la prevalencia de la depresión postparto en madres indias. En la búsqueda se utilizaron epígrafes temáticos y palabras clave sin restricciones de lenguaje. La calidad se evaluó con la escala de evaluación de calidad de Newcastle‒Ottawa. Se realizó un metaanálisis utilizando un modelo de efectos aleatorios. El análisis y la metarregresión de los subgrupos se realizaron con fines de heterogeneidad y se utilizó la prueba de Egger para evaluar las tendencias de las publicaciones.

**Resultados:**

Se analizaron treinta y ocho estudios que incluían 20 043 mujeres. Los estudios tuvieron un alto grado de heterogeneidad (I2 = 96,8%) y se encontraron pruebas de tendencias de publicaciones (tendencia de Egger = 2,58; intervalo de confianza, CI, del 95%: 0,83–4,33). La estimación general calculada sobre la prevalencia de la depresión posparto fue del 22% (IC del 95%: 19–25). La prevalencia obtenida fue del 19% (IC del 95%: 17–22), salvo en 8 estudios que informaron de depresión posparto dentro de las 2 primeras semanas después del parto. Se descubrieron pequeñas diferencias con poca importancia en la prevalencia obtenida según la edad de la madre, la ubicación geográfica y el marco del estudio. Los factores de riesgo descubiertos sobre la depresión posparto incluían dificultades financieras, violencia doméstica, historial pasado de enfermedad psiquiátrica, conflicto marital, ausencia de apoyo por parte del marido y nacimiento de una niña.

**Conclusión:**

El análisis muestra una alta prevalencia de depresión posparto en madres indias. Es necesario asignar más recursos para aumentar la capacidad de la atención de salud mental de las madres en la India.

## ملخص

**الغرض:**

توفير تقدير للعبء الناتج عن الإصابة باكتئاب ما بعد الولادة بين الأمهات الهنديات والنظر في بعض عوامل الخطورة لتلك الحالة.

**الطريقة:**

لقد بحثنا في قواعد بيانات PubMed®‎ وGoogle Scholar وEmbase®‎ عن مقالات نشرت من عام 2000 حتى 31 مارس/آذار 2016 عن انتشار الإصابة باكتئاب ما بعد الولادة بين الأمهات الهنديات. وقد استخدم البحث عناوين الموضوع والكلمات الرئيسية دون قيود لغوية. وتم تقييم الجودة النوعية من خلال مقياس Newcastle‒Ottawa لتقييم الجودة النوعية. كما قمنا بإجراء تحليل تَلوي مستخدمين نموذجًا للمؤثرات العشوائية. وتم إجراء تحليل المجموعة الفرعية والتحوف التلوي لعدم التجانس، وتم استخدام اختبار Egger لتقييم عامل التحيز في نشر البحوث العلمية.

**النتائج:**

تم تحليل 38 دراسة شملت 20043 امرأة. وكانت الدراسات على درجة عالية من عدم التجانس (حيث بلغ مربع معامل عدم التجانس I2: = ‏96.8‏٪) وكان هناك دليل على وجود تحيز في المنشورات العلمية (حيث بلغ مقدار التحيز وفقًا لمعيار Egger‏ = 2.58؛ بنطاق ثقة بنسبة 95‏٪: 0.83–4.33). وكان التقدير المجمع الكلي لمعدل انتشار الاكتئاب بعد الولادة ‏‏22٪ (بنطاق ثقة مقداره ‏95‏٪: 19–25). وباستثناء ثماني دراسات، فإن التقارير المتعلقة باكتئاب ما بعد الولادة في غضون أسبوعين من الولادة قد حققت انتشارًا مجمعًا بنسبة ‏‏‏19٪ (بنطاق ثقة مقداره ‏95‏٪: 17–22). ووُجدت فروق صغيرة ولكن غير ملموسة في الانتشار المجمع تبعًا لعمر الأم، والموقع الجغرافي، ومحيط الدراسة. وشملت عوامل الخطر المبلغ عنها لاكتئاب ما بعد الولادة الصعوبات المالية، ووجود العنف المنزلي، والتاريخ السابق للأمراض النفسية في الأم، والصراع الزوجي، ونقص الدعم من الزوج، وولادة طفلة أنثي.

**الاستنتاج:**

أظهرت المراجعة معدلاً مرتفعًا لانتشار اكتئاب ما بعد الولادة بين الأمهات الهنديات. ويلزم تخصيص مزيد من الموارد لبناء القدرات في مجال الرعاية الصحية العقلية للأمهات في الهند.

## 摘要

**目的:**

对印度产妇产后抑郁症负担进行估计，调查此情况下的风险因素。

**方法:**

我们检索了 PubMed®、Google 学术 (Google Scholar) 和 Embase® 数据库中从 2000 年至 2016 年 3 月 31 日发布的关于印度产妇产后抑郁症患病率的文章。 检索采用没有语言局限性的主题词和关键词。 通过纽卡斯尔-渥太华质量评估量表评估质量。 我们使用随机效应模型进行元分析。 对异质性进行分组分析和元回归分析，并采用 Egger 测试来评估发布偏倚。

**结果:**

我们分析了涉及 20043 名女性的 38 项研究。 研究具有高异质性（I2 = 96.8％），有证据显示存在发布偏差（Egger 偏差 = 2.58；95％ 置信区间，CI： 0.83–4.33). 产后抑郁症患病率的汇总估计值为 22％ （95％ CI： 19–25). 排除报告称在产后 2 周内患有产后抑郁症的 8 项研究后的汇总患病率为 19％（95％ CI： 17–22)。 不同产妇年龄、地理位置和研究背景会出现细微（非显著）差别。 据报告，产后抑郁症的风险因素包括经济困难、家庭暴力、产妇精神病史、夫妻矛盾、缺乏丈夫的支持和生出女婴。

**结论:**

本次考察显示印度产妇产后抑郁症患病率很高。 需要为印度产妇精神健康护理的能力建设配置更多资源。

## Резюме

**Цель:**

Дать оценку бремени послеродовой депрессии у матерей в Индии и изучить некоторые факторы риска для этого состояния.

**Методы:**

Авторы провели поиск в базах данных PubMed®, Google Scholar и Embase® на предмет статей, опубликованных с 2000 года по 31 марта 2016 года, которые посвящены распространенности послеродовой депрессии у матерей в Индии. При поиске использовались предметные указатели и ключевые слова без языковых ограничений. Качество исследований оценивалось по шкале оценки качества Ньюкасл-Оттава. Авторы провели метаанализ с использованием модели со случайными эффектами. Для определения гетерогенности проводился анализ данных в подгруппах и метарегрессия. Кроме того, с помощью теста Эггера была выполнена оценка на предмет систематической ошибки, связанной с предпочтительной публикацией положительных результатов исследования (публикационная ошибка).

**Результаты:**

Был проведен анализ тридцати восьми исследований, в которых принимали участие 20 043 женщины. Исследования имели высокую степень гетерогенности (I2 = 96,8%), также имелись признаки систематической публикационной ошибки (отклонение Эггера = 2,58, 95% доверительный интервал, ДИ: 0,83–4,33). Согласно объединенной оценке с использованием всех имеющихся данных, распространенность послеродовой депрессии составила 22% (95% ДИ: 19–25). После исключения 8 исследований, сообщающих о случаях послеродовой депрессии в течение 2 недель после родов, объединенная распространенность составила 19% (95% ДИ: 17–22). Различия в объединенной распространенности, обусловленные возрастом матерей, географическим расположением и условиями проведения исследования, были незначительны. Выявленные факторы риска для послеродовой депрессии включали: финансовые трудности, домашнее насилие, историю психических заболеваний у матери, супружеские конфликты, отсутствие поддержки от мужа и рождение ребенка женского пола.****

**Вывод:**

Обзор свидетельствует о высокой распространенности послеродовой депрессии у матерей в Индии. Необходимо выделить больше ресурсов для создания потенциала в области охраны психического здоровья матерей в Индии.****

## Introduction

Postpartum psychiatric disorders can be divided into three categories: postpartum blues; postpartum psychosis and postpartum depression.^1^^,^^2^ Postpartum blues, with an incidence of 300‒750 per 1000 mothers globally, may resolve in a few days to a week, has few negative sequelae and usually requires only reassurance.^1^ Postpartum psychosis, which has a global prevalence ranging from 0.89 to 2.6 per 1000 births, is a severe disorder that begins within four weeks postpartum and requires hospitalization.^3^ Postpartum depression can start soon after childbirth or as a continuation of antenatal depression and needs to be treated.^1^ The global prevalence of postpartum depression has been estimated as 100‒150 per 1000 births.^4^

Postpartum depression can predispose to chronic or recurrent depression, which may affect the mother‒infant relationship and child growth and development.^1^^,^^5^^–^^7^ Children of mothers with postpartum depression have greater cognitive, behavioural and interpersonal problems compared with the children of non-depressed mothers.^5^^,^^6^ A meta-analysis in developing countries showed that the children of mothers with postpartum depression are at greater risk of being underweight and stunted.^6^ Moreover, mothers who are depressed are more likely not to breastfeed their babies and not seek health care appropriately.^5^ A longitudinal study in a low- and middle-income country documented that maternal postpartum depression is associated with adverse psychological outcomes in children up to 10 years later.^8^ While postpartum depression is a considerable health issue for many women, the disorder often remains undiagnosed and hence untreated.^1^^,^^9^

The current literature suggests that the burden of perinatal mental health disorders, including postpartum depression, is high in low- and lower-middle-income countries. A systematic review of 47 studies in 18 countries reported a prevalence of 18.6% (95% confidence interval, CI: 18.0‒19.2).^10^ Scarcity of available mental health resources,^11^ inequities in their distribution and inefficiencies in their utilization are key obstacles to optimal mental health, especially in lower resource countries. Addressing these issues is therefore a priority for national governments and their international partners. The impetus for this will come from reliable scientific evidence of the burden of mental health problems and their adverse consequences. 

Despite the launch of India’s national mental health programme in 1982, maternal mental health is still not a prominent component of the programme. Dedicated maternal mental health services are largely deficient in health-care facilities, and health workers lack mental health training. The availability of mental health specialists is limited or non-existent in peripheral health-care facilities.^12^ Furthermore, there is currently no screening tool designated for use in clinical practice and no data are routinely collected on the proportion of perinatal women with postpartum depression.^12^

India is experiencing a steady decline in maternal mortality,^13^ which means that the focus of care in the future will shift towards reducing maternal morbidity, including mental health disorders. Despite the growing number of empirical studies on postpartum depression in India, there is a lack of robust systematic evidence that looks not only at the overall burden of postpartum depression, but also its associated risk factors. Our current understanding of the epidemiology of postpartum depression is largely dependent on a few regional studies, with very few nationwide data. The current review was done to fill this gap, by providing an updated estimate of the burden of postpartum depression in India, to synthesize the important risk factors and to provide evidence-based data for prioritization of maternal mental health care.

## Methods

### Data sources and search strategy

Two authors (RPU and AP) independently searched PubMed®, Google Scholar and Embase® databases for articles on the prevalence of postpartum depression in India, published until 31 March 2016. The search strategy (Box 1) used subject headings and keywords with no language restrictions. Any discrepancy in the search results was planned to be discussed with a third author (AKR). We also searched the bibliographies of included articles and government reports on government websites to identify relevant primary literature to be included in the final analysis. For studies with missing data or requiring clarification, we contacted the principal investigators.

Box 1 **Search keywords used for identification of articles for the review of the prevalence of postpartum depression, India, 2000–2015**

1. (“depression” OR “depressive disorder” OR “blues” OR “distress” OR “bipolar” OR “bi-polar” OR “mood disorder” OR “anxiety disorder”)
2. (“postpartum” OR “postnatal” OR “perinatal” OR “post birth” OR “after delivery” OR “after birth” OR “puerperium” OR “puerperal”)
3. (“prevalence” OR “incidence” OR “burden” OR “estimate” OR “epidemiology”)
4. (“India” OR “South East Asia”)
5. (#1 AND #2 AND #3 AND #4)
6. (Addresses[ptyp] OR Autobiography[ptyp] OR Bibliography[ptyp] OR Biography[ptyp] OR pubmed books[filter] OR Case Reports[ptyp] OR Congresses[ptyp] OR Consensus Development Conference[ptyp] OR Directory[ptyp] OR Duplicate Publication[ptyp] OR Editorial[ptyp] OR Systematic reviews OR Meta analysis OR Festschrift[ptyp] OR Guideline[ptyp] OR In Vitro[ptyp] OR Interview[ptyp] OR Lectures [ptyp] OR Legal Cases[ptyp] OR News[ptyp] OR Newspaper Article[ptyp] OR Personal Narratives [ptyp] OR Portraits[ptyp] OR Retracted Publication[ ptyp] OR Twin Study[ptyp] OR Video-Audio Media[ptyp])
7. (#5 NOT #6) Filters: Original research; published in the past 15 years; humans

### Study selection and data extraction

For a study to be included in the systematic review, it had to be original research done in India, within a cross-sectional framework of a few weeks to 1 year post-birth. We excluded research done in a specific population, such as mothers living with human immunodeficiency virus; research including mothers with any current chronic disease. To have a fairly recent estimate of the burden of postpartum depression, we considered only studies published from the year 2000 and later. After initial screening of titles and abstracts, we reviewed the full text of eligible publications. Decisions about inclusion of studies and interpretation of data were resolved by discussion among the reviewers. Data from all studies meeting the inclusion criteria were extracted and tabulated.

### Study quality assessment

We used the Newcastle‒Ottawa quality assessment scale adapted for cross-sectional studies.^14^^,^^15^ The scale is used to score the articles under three categories: (i) selection (score 0‒5); (ii) comparability (score 0‒2 ); and (iii) outcome (score 0‒3); total score range 0‒10. The selection category consists of parameters, such as representativeness of the sample, adequacy of the sample size, non-response rate and use of a validated measurement tool to gather data on exposure. The comparability category examines whether subjects in different outcome groups are comparable based on the study design and analysis and whether confounding factors were controlled for or not. The outcome category includes whether data on outcome(s) were collected by independent blind assessment, through records or by self-reporting. The outcome category also includes whether the statistical tests used to analyse data were clearly described and whether these tests were appropriate or not. Two authors (RPU and KS) made separate quality assessments of the included studies. In case of any discrepancy, a third author (AP) was consulted. We grouped the studies into those with quality scores ≤ 5 and > 5.

### Data analysis

We did a meta-analysis of the reported prevalence of postpartum depression in the included studies. Heterogeneity between studies was quantified by the *I*^2^ statistic. We considered *I*^2^ values > 50% to represent substantial heterogeneity.^16^ The degree of heterogeneity among the studies was high (> 95%), and thus we used a random effects model to derive the pooled estimate for postpartum depression in mothers. The final estimates of prevalence were reported as percentages with 95% CI.

We did a subgroup analysis by excluding articles in which depression was assessed within 2 weeks postpartum,^1^^,^^17^^,^^18^ since some researchers argue that it is difficult to differentiate postpartum depression from postpartum blues within 2 weeks of birth. In addition, the Edinburgh postnatal depression scale, which was used in the majority of studies we identified, can give false-positive results in the early postpartum period.

We also did separate subgroup analyses on each of the following factors: place of study (geographical location; rural or urban; hospital or community); study instrument used; quality score of the articles; time of publication; and age of mothers. Not all the studies provided data on the mean age of the study participants that was required for subgroup analysis; however, the proportion of mothers in specific age ranges were available. Using this information, we estimated the mean age of the study participants. For studies that reported the prevalence of postpartum depression in mothers at different time points, we used the prevalence reported in the earliest time point to reduce the effect of lost to follow-up. We used meta-regression analysis to identify factors contributing to the heterogeneity in effect size, i.e. the pooled proportion of mothers with postpartum depression.

We assessed publication bias with the Egger test and used a funnel plot to graphically represent the bias. Finally, we listed the risk factors for postpartum depression. We used Stata software, version 14 (StataCorp. LLC, College Station, United States of America) for all analyses.

## Results

### Characteristics of the studies

**Fig. 1:**
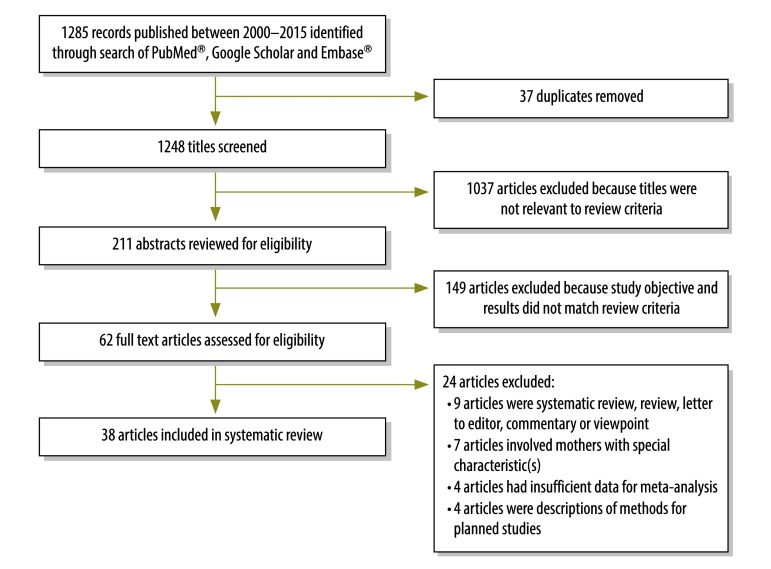
**Flowchart showing the selection of studies for the systematic review of the prevalence of postpartum depression, India, 2000–2015**

Of the 1285 articles we identified in our search, we screened 1248 titles of unique articles. Out of these, we reviewed 211 relevant abstracts, assessed 62 full-text articles for eligibility and included 38 articles in our final analysis.^19^^–^^56^ (Fig 1). These 38 studies included data from 20 043 mothers in total. More of the articles (26 studies) were published in the most recent five-year period 2011‒2015 than in the earlier periods 2000–2005 (6) and 2006–2010 (6). The majority of studies were from south India (16 studies), followed the western (9) and northern regions (7) of the country; 24 studies were done in an urban setting and 29 in hospitals (Table 1; available at: http://www.who.int/bulletin/volumes/94/10/17-192237). In 19 studies, the mean age of the study mothers was ≤ 25 years. The Edinburgh postnatal depression scale was the most commonly used study instrument (29 studies). The median quality score for the studies was 5 (21 articles had a score of ≤ 5 and 17 had a score >  5). 

**Table 1:** Characteristics of the studies identified in the systematic review of the prevalence of postpartum depression in mothers, India, 2000–2015

Study	Place of study (region)	Study setting	Study design	Study instrument	Mean age of participants, years (SD)	Timing of data collection postpartum	No. of women	No. of mothers with depression	Quality score^a^
Affonso et al., 2000^56^	Kolkata (east)	NR	Cross-sectional	EPDS	> 25^b^	At 1-2 weeks	110	39	6
						At 4-6 weeks	102	33	
				BDI		At 1-2 weeks	106	35	
						At 4-6 weeks	101	25	
Patel et al., 2002^55^	Goa (south-west)	Urban hospital	Cohort	EPDS	26 (4)	At 6–8 weeks	252	59	8
						At 6 months	235	51	
Chandran et al., 2002^54^	Tamil Nadu (south)	Rural community	Cohort	CIS-R	22.8 (3.7)	At 6–12 weeks	301	33	8
Patel et al., 2003^53^	Goa (south-west)	Urban hospital	Cohort	EPDS	26 (NR)	At 6–8 weeks	171	37	7
Sood & Sood, 2003^52^	Uttar Pradesh (north)	Urban hospital	Cohort	BDI	24 (3)	At 3–7 days	75	15	4
						At 4–6 weeks	70	9	
Prabhu et al., 2005^51^	Tamil Nadu (south)	Not clearly defined	Cross-sectional	EPDS	NR	At 3–4 weeks	478	28	5
Kalita et al., 2008^50^	Assam (North east)	Urban hospital	Cross-sectional	EPDS	25.1 (4.7)	At 6 weeks	100	18	4
Nagpal et al., 2008^49^	Delhi (north)	Urban community	Cross-sectional	EPDS	27 (25.8–28.2)^c^	Within 6 months	172	63	8
Mariam & Srinivasan, 2009^48^	Karnataka (south)	Urban hospital	Cohort	EPDS	23.9 (3.6)	Within 6–10 weeks	132	39	3
Ghosh & Goswami, 2009^47^	Kolkata (east)	Urban hospital	Cross-sectional	EPDS	25.3 (NR)	At 4–7 days	6000	1505	2
Savarimuthu et al., 2010^46^	Tamil Nadu (south)	Rural community	Cross-sectional	EPDS	23.6 (3.4)	At 2–10 weeks	137	36	7
Sankapithilu et al., 2010^45^	Mysore (south)	Urban hospital	Cross-sectional	EPDS	23.8 (NR)	Within 3 months	100	30	5
Manjunath et al., 2011^44^	Karnataka (south)	Urban hospital	Cross-sectional	EPDS	18–45^d^	Within 2 weeks	123	72	5
Iyengar et al., 2012^43^	Rajasthan (west)	Rural community	Cohort	EPDS	26.4 (NR)	At 6–8 weeks	430	87	9
						At 12 months	275	32	
Prost et al., 2012^42^	Jharkand; Orrisa (east)	Rural community	Control arm of a clustered RCT	Kessler 10--item scale	25.5 (5.3)	At 6 weeks	5801	669	9
Dubey et al., 2012^41^	Delhi (north)	Urban hospital	Cross-sectional	EPDS	24.3 (3.2)	Day 1 to week 1	293	18	3
Hegde et al. 2012^40^	Karnataka (south)	Urban hospital	Cross-sectional	MINI with DSM-IV criteria	24.3 (7.9)	At 2–3 days	150	17	9
						At 6 weeks	139	22	
						At 14 weeks	129	20	
Desai et al., 2012^39^	Gujarat (west)	Urban hospital	Cross-sectional	Semi-structured interview based on DSM-IV-TR criteria	23.8 (NR)	Up to 1 year	200	25	4
Gokhale et al., 2013^38^	Gujarat (west)	Urban hospital	Cross-sectional	EPDS	25.2 (NR)	At day 1	200	22	3
						At day 6	108	8	
						At week 6	62	2	
Sudeepa et al., 2013^37^	Bangalore (south)	Rural hospital	Cross-sectional	EPDS	22.6 (2.4)	At 6–8 weeks	244	28	3
Prakash et al., 2013^36^	Gujarat (west)	Urban hospital	Cross-sectional	EPDS	NR	Within 24 hours	155	50	2
Gupta et al., 2013^35^	Delhi (north)	Urban hospital	Cross-sectional	PRIME-MD	24.6 (3.7)	At 6 weeks	202	32	9
Dhiman et al., 2014^34^	Puducherry (south)	Urban hospital	Cross-sectional	EPDS	NR	At 24–48 hours	103	58	2
Jain et al., 2014^33^	Delhi (north)	Urban hospital	Cross-sectional	EPDS	26.3 (NR)	Within 1 week	1537	105	7
Saldanha et al., 2014^32^	Maharashtra (west)	Urban hospital	Cross-sectional	EPDS	24.9 (NR)	At 6 weeks	186	40	5
Dhande et al., 2014^31^	Wardha (west)	Rural hospital	Cross-sectional	EPDS	24.3 (NR)	Within 6 months	67	16	5
Poomalar & Arounassalame, 2014^30^	Puducherry (south)	Urban hospital	Cross-sectional	EPDS	25.6 (NR)	Within 1 week	254	26	6
Johnson et al., 2015^29^	Karnataka (south)	Rural hospital	Cross-sectional	EPDS	23.2 (NR)	Within 1 week	74	33	7
						At 6–8 weeks	49	23	
Patel et al., 2015^28^	Gujarat (west)	Urban hospital	Cross-sectional	EPDS	25.2 (4.2)	Within 1 week	134	65	3
Hiremath et al., 2015^27^	Maharashtra (west)	Urban hospital	Cross-sectional	EPDS	29.3 (NR)	Within 6 weeks	80	13	4
Hirani & Bala, 2015^26^	Gujarat (West)	Rural community	Cross-sectional	EPDS	23.3 (NR)	At 1–6 weeks	516	62	4
Bodhare et al., 2015^25^	Telengana (south)	Urban hospital	Cross-sectional	PHQ-9	23.2 (3.2)	At 6–8 weeks	274	109	8
Kolisetty & Jyothi, 2015^24^	Karnataka (south)	Urban hospital	Cross-sectional	DSM-IV	28.2 (NR)	Within 6 weeks	100	22	6
Srivastava et al. 2015^23^	Uttar Pradesh (north)	Urban hospital	Cross-sectional	DSM-IV-TR	25.1 (NR)	Within 4 weeks	100	16	1
Kumar et al., 2015^22^	Karnataka (south)	Rural hospital	Cross-sectional	EPDS	22.7 (3.3)	At 6−8 weeks	310	43	8
Suguna et al., 2015^21^	Bangalore (south)	Rural hospital	Cross-sectional	EPDS	23.6 (NR)	Within 6 weeks	180	32	1
Shrestha et al., 2015^20^	Haryana (north)	Rural community	Cross-sectional	EPDS	22.6 (NR)	At 6 weeks	200	24	5
Shivalli & Gururaj, 2015^19^	Karnataka (south)	Rural hospital	Cross-sectional	EPDS	23.1 (2.9)	At 4–10 weeks	102	32	9

BDI: Beck depression inventory; CIS-R: clinical interview schedule-revised; DSM-IV: diagnostic and statistical manual of mental disorders 4th edition; DSM-IV-TR: “text revision” of diagnostic and statistical manual of mental disorders 4th edition; EPDS: Edinburgh postnatal depression scale; MINI: M.I.N.I. international neuropsychiatric interview; NR: not reported; PHQ-9: 9-item patient health questionnaire; PRIME-MD: primary care evaluation of mental disorders; RCT: randomized controlled trial; SD: standard deviation.

^a^ We used the Newcastle–Ottawa quality assessment scale with a maximum score of 10.^14^

^b^ Reported average age of participants > 25 years.

^c^ Range is 95% confidence interval.

^d^ Range of ages.

### Prevalence of postpartum depression

Based on the random effects model, the overall pooled estimate of the prevalence of postpartum depression in Indian mothers was 22% (95% CI: 19–25; Fig. 2). Eight studies included women reporting depression within 2 weeks of delivery. After excluding these, the pooled prevalence for the remaining 30 studies (11 257 women) was 19% (95% CI: 17–22; Fig. 3).

**Fig. 2:**
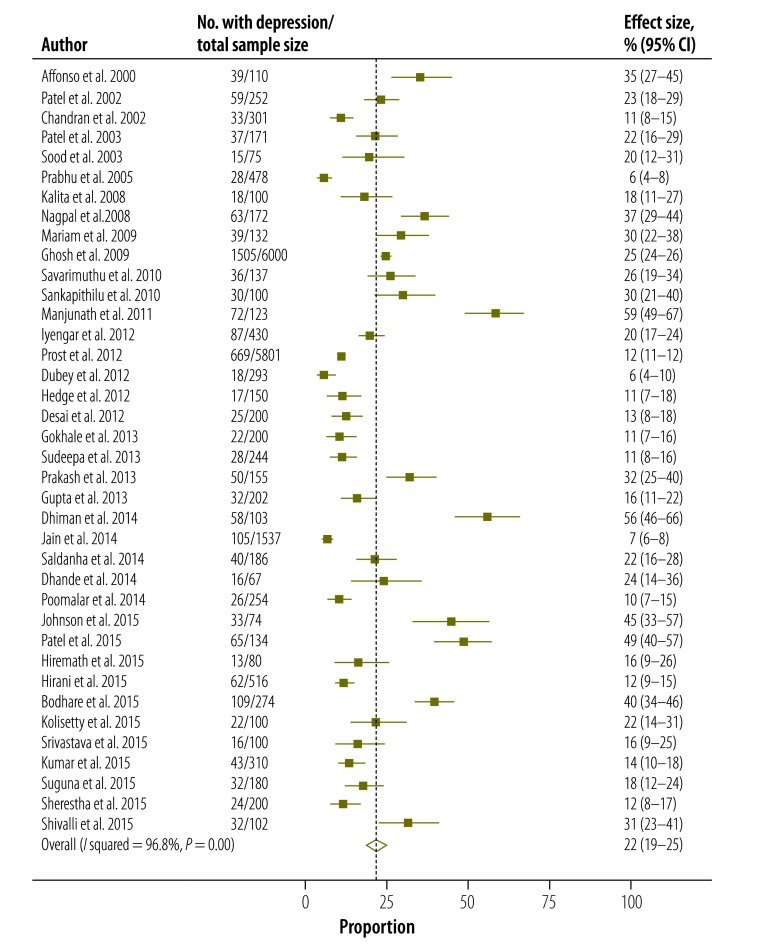
**Estimated prevalence of postpartum depression, pooling all selected studies (*n* = 38), India, 2000–2015**

CI: confidence interval.

Notes: Results from random effect analysis. Studies included a total of 20 043 women. The dashed line passing through the midpoint of the diamond denotes the point estimate of the overall pooled effect size and the lateral tips of the diamond represent 95% confidence intervals.

**Fig. 3:**
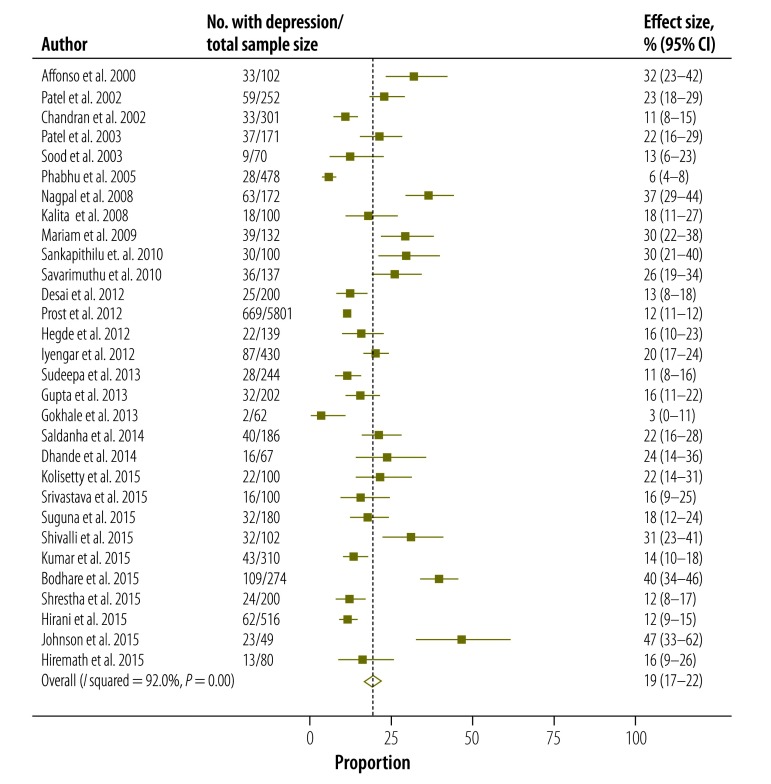
**Estimated prevalence of postpartum depression after excluding studies reporting depression within 2 weeks postpartum (n=30), India, 2000-2015**

CI: confidence interval.

Notes: Results from random effect analysis. Studies included a total of 11 257 women. The dashed line passing through the midpoint of the diamond denotes the point estimate of the overall pooled effect size and the lateral tips of the diamond represent 95% confidence intervals.

**Fig. 4:**
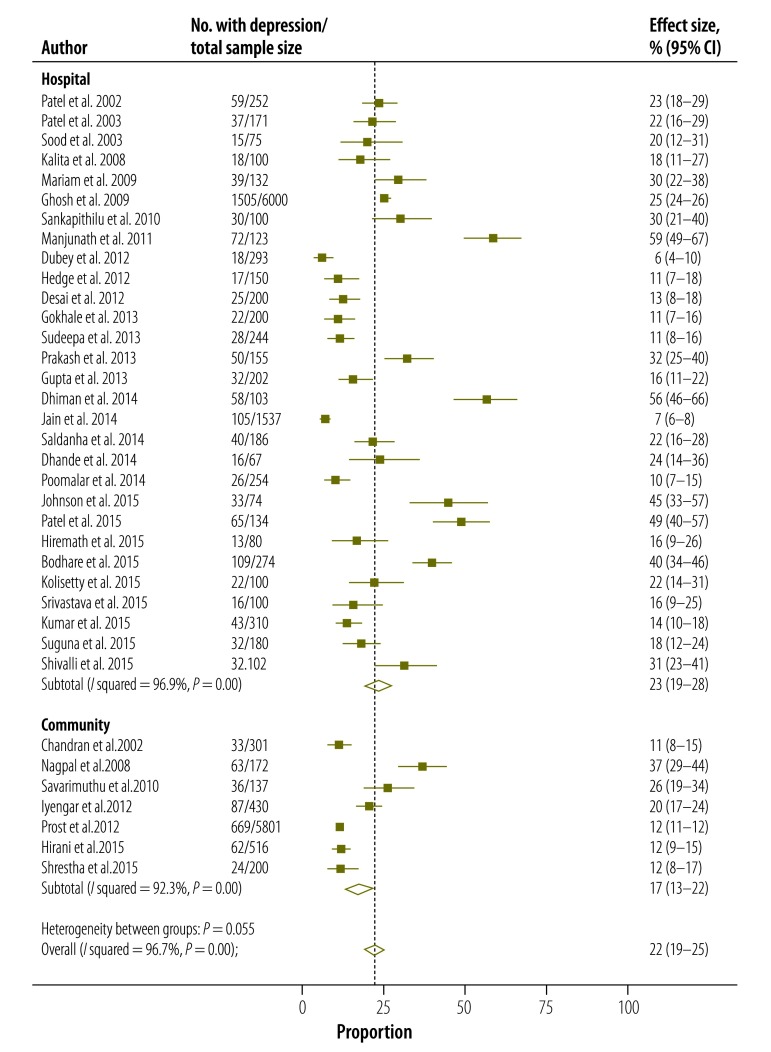
**Estimated prevalence of postpartum depression from hospital- and community-based studies (*n* = 36), India, 2000–2015**

The estimated overall pooled prevalence was highest in the southern region of the country (26%; 95% CI: 19–32), followed by eastern (23%; 95% CI: 12-35), south-western (23%; 95% CI: 19–27) and western regions (21%; 95% CI: 15–28; Table 2). The northern region of India had the lowest prevalence (15%; 95% CI: 10–21). The pooled prevalence was higher, but not significantly so, for studies conducted in hospital settings (23%; 95% CI: 19–28) than in community settings (17%; 95% CI: 13–22); Fig. 4; Table 2) and in urban versus rural areas (24%; 95% CI: 19–29 versus 17%; 95% CI: 14–21). Prevalence was 20% (95% CI: 16–24) and 21% (95% CI: 16–26) when studies with mean maternal age of ≤ 25 years and > 25 years were pooled respectively.

**Table 2:** Subgroup analysis in the systematic review of the prevalence of postpartum depression, India, 2000–2015

Study characteristic	No. of women	No. of studies	Pooled prevalence, % (95% CI)	*P*	*P* for meta-regression
**All**	20 043	38	22 (19–25)		
**Region**					
East	11911	3	23 (12-35)	< 0.05	0.63
West	1 968	9	21 (15–28)		0.66
North	2 579	7	15 (10–21)		0.20
South	3 062	16	26 (19–32)		Ref.
North-east	100	1	18 (10–26)		0.81
South-west	423	2	23 (19–27)		0.70
**Setting**^a^					
Hospital	11 898	29	23 (19–28)	< 0.05	Ref.
Community	7 557	7	17 (13–22)		0.41
**Area^a^**					
Urban	11 093	24	24 (19–29)	< 0.05	Ref.
Rural	8 362	12	17 (14–21)		0.16
**Study instrument**					
EPDS	12 840	29	24 (20–28)	< 0.05	Ref.
Others^b^	7 203	9	17 (13–22)		0.22
**Weeks postpartum **					
≥ 2	11 257	30	19 (17–22)	< 0.05	Ref.
< 2	8 599**^c^**	8	30 (20–39)		0.29
**Age of participants, years^d^**					
≤ 25	3 743	19	20 (16–24)	< 0.05	Ref.
> 25	15 441	15	21 (16–26)		0.25
**Study quality score**					
≤ 5	9 666	21	22 (18–27)	< 0.05	Ref.
> 5	10 377	17	21 (18–25)		0.59
**Publication year**					
2000–2005	1 387	6	19 (11–27)	< 0.05	0.91
2006–2010	6 641	6	27 (23–32)		0.89
2011–2015	12 015	26	21 (18–24)		Ref.

CI: confidence interval; EPDS: Edinburgh postnatal depression scale; Ref.: reference category.

^a^ Prabhu^51^ et al. and Affonso et al.^56^ did not provide information on study setting.

^b^ Includes diagnostic and statistical manual of mental disorders 4th edition (DSM-IV); 9-item patient health questionnaire; primary care evaluation of mental disorders; Beck depression inventory; M.I.N.I. international neuropsychiatric interview plus DSM-IV; Kessler 10-item scale; and clinical interview schedule‒revised.

^c^ Numbers do not total 20 043 as the number of women varies according to the time of assessment postpartum.

^d^ Dhiman et al.,^34^ Prakash et al.,^36^ Manjunath et al.^44^ and Prabhu et al.^51^ either did not provide the age of mothers or sufficient data for the analysis.

CI: confidence interval.

Notes: Results from random effect analysis. Studies included a total of 19 455 women (11 898 in hospital-based studies and 7557 in community-based studies). Two studies (Prabhu^51^ et al. and Affonso et al.^56^) did not provide information on study setting. The dashed line passing through the midpoint of the diamond denotes the point estimate of the overall pooled effect size and the lateral tips of the diamond represent 95% confidence intervals. The three diamonds from the top represent the pooled estimate for hospital-based studies, community-based studies and overall pooled estimate respectively.

Pooling of studies that used the Edinburgh postnatal depression scale as the study instrument produced a prevalence of 24% (95% CI: 20–28) compared with 17% (95% CI: 13–22) in those that used other study instruments (Table 2). 

Studies with a quality score ≤ 5 had a pooled prevalence of 22% (95% CI: 18–27) and those with a score > 5 had a prevalence of 21% (95% CI: 18–25).

**Fig. 5:**
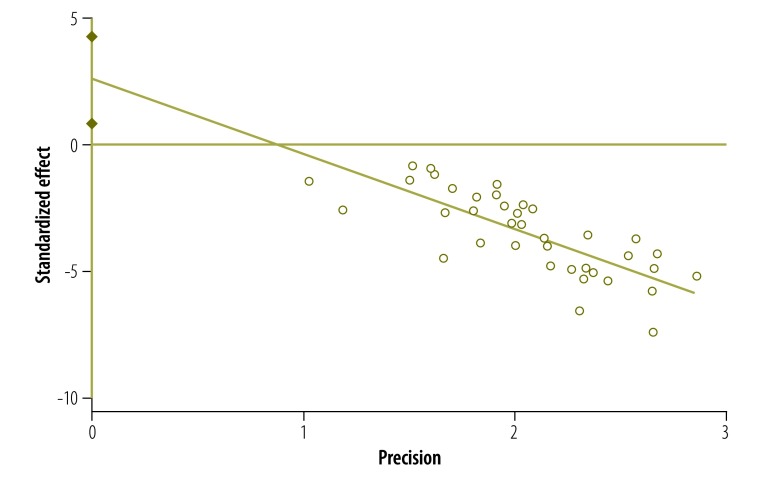
**Egger plot for publication bias in the meta-analysis of studies (*n* = 38) on the prevalence of postpartum depression, India, 2000–2015**

**Fig. 6:**
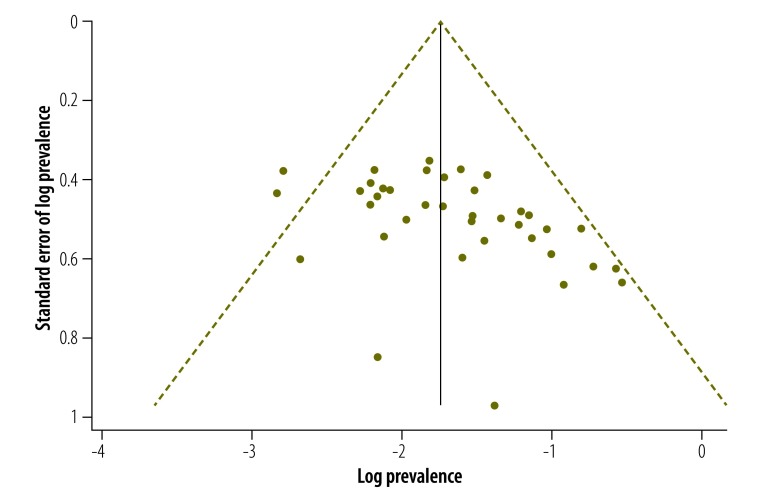
**Funnel plot of publication bias in the meta-analysis of studies (*n* = 38) on the prevalence of postpartum depression, India, 2000–2015.**

Notes: The outer dashed lines indicate the triangular region within which 95% of studies are expected to lie in the absence of both biases and heterogeneity. The solid line represents the log of the total overall estimate of the meta-analysis.

The studies had a high degree of heterogeneity (*I*^2^ = 96.8%). Both the Egger plot (Egger bias = 2.58; 95% CI: 0.83–4.33; Fig. 5) and the funnel plot (Fig. 6) showed evidence of publication bias.

### Risk factors

A total of 32 studies reported risk factors for postpartum depression. The risk factors most commonly reported were financial difficulties (in 19 out of 21 studies that included this variable), domestic violence (6/8 studies), past history of psychiatric illness in the mother (8/11 studies), marital conflict (10/14 studies), lack of support from the husband (7/11 studies) and birth of a female baby (16/25 studies). Other commonly reported risk factors were lack of support from the family network (8/14 studies), recent stressful life event (6/11 studies), family history of psychiatric illness (7/13 studies), sick baby or death of the baby (6/13 studies) and substance abuse by the husband (4/9 studies). Preterm or low birth-weight baby, high parity, low maternal education, current medical illness, complication in current pregnancy and unwanted or unplanned pregnancy and previous female child, were some of the other reported risk factors (Table 3).

**Table 3:** Risk factors for postpartum depression reported by studies included in the systematic review, India, 2000–2015

Variable	No. of studies	
	Total	Reporting risk for postpartum depression
**Individual factors**		
High maternal age^a^	28^b^	4
Low maternal age^a^	28^b^	3
Low maternal education	27^c^	10
Current medical illness	6	2
Past history of psychiatric illness, anxiety or low mood	11	8
Family history of psychiatric illness	13	7
Recent stressful life event	11	6
Low self-esteem	4	2
**Husband & marital relationship factors**		
Marital conflict	14	10
Domestic violence	8	6
Lack of support from husband	11	7
Addiction in husband	9	4
Financial difficulties	21	19
**Pregnancy-related factors**		
Unplanned or unwanted pregnancy	14	4
Past history of obstetric complication	18	3
Complicated or eventful current pregnancy	22	8
Female child born in the current pregnancy	25	16
Previous female child	14	4
Primigravida	23	4
High parity	23	9
Mood swings during pregnancy	12	4
Caesarean section	15	5
Preterm or low-birth-weight baby	16	5
Sickness or death of baby	13	6
**Other psychological factors**		
Conflict with in-laws	11	3
Lack of support from family networks	14	8
Lack of confidant/close friend	12	2

^a^ High maternal age reported as > 30–35 years. Low maternal age reported as < 25 years.

^b^ Total number of studies that analysed maternal age as a risk factor for postpartum depression.

^c^ Studies that analysed maternal education as a risk factor for postpartum depression.

## Discussion

The pooled prevalence of postpartum depression in India in our meta-analysis was 22% (95% CI: 19–25). A systematic review of studies in 11 high-income countries showed that, based on point prevalence estimates, around 12.9% (95% CI: 10.6–15.8) of mothers were depressed at three months postpartum.^57^ Data from 23 studies conducted in low- and middle-income countries, which included 38 142 women, was 19.2% (95% CI: 15.5–23.0).^58^ Another systematic review from 34 studies found that the prevalence of common mental disorders in the postpartum period in low- and lower-middle income countries was 19.8% (95% CI: 19.2–20.6).^10^ These estimates in low- and middle-income countries are similar to ours and, taken together, they support an argument for placing greater importance on maternal mental health as part of overall efforts to improve maternal and child health.

Although facility-based deliveries are increasing in many low- and middle-income countries, a high proportion of pregnant mothers still deliver at home.^59^ Beyond the lack of awareness of postpartum depression by health professionals, there are issues that may be barriers to prompt recognition and management of the illness.^60^^–^^62^ In India, women who deliver at a health facility often stay for less than 48 hours after delivery.^63^ This leaves little opportunity for health personnel to counsel the mother and family members on the signs and symptoms of postpartum depression and when to seek care. In low- and middle-income countries, the proportion of women who visit the health facility for postpartum visits is generally low and consequently mental disorders often remain undetected and unmanaged, especially for those delivering at home.^64^ Analysis of demographic and health survey data from 75 countdown countries showed that postnatal care visits for mothers have low coverage among interventions on the continuum of maternal and child care^65^ Postnatal traditions, such as the period of seclusion at home observed in many cultures, can negatively affect care-seeking behaviour in the postpartum period. Furthermore, mothers may be reluctant to admit their suffering either because of social taboos associated with depression or concerns about being labelled as a mother who failed to deliver the responsibilities of child care. In the current public health system in most low- and middle-income countries, including India, primary-care workers are supposed to be in regular contact with recently delivered mothers. However, at postnatal visits community health workers tend to focus on promoting essential infant care practices, with lower priority given to the mother’s health.^63^^,^^66^ These factors might explain, to some extent, the lack of availability of reliable, routine data on the burden of postpartum depression in low- and middle-income countries.

A strength of our study is the large sample of recently delivered mothers included in the review. This is probably the first review that documents the overall estimated prevalence of postpartum depression in India. The study has its limitations as well. Most of the studies included in the review did not provide effect sizes against the risk factors for postpartum depression and this precluded pooling of risk factors to provide an estimate. Most of the studies included in the review used the Edinburgh postnatal depression scale and the cut-offs used to label postpartum depression varied among studies. This could limit the internal validity of our findings. We observed significant heterogeneity in the results and performed subgroup analysis and meta-regression. The meta-regression analysis was able to explain < 10% of the heterogeneity and suggests that unidentified factors were causing such heterogeneity. 

Among the studies included in our review, risk factors for postpartum depression included financial difficulties, birth of a female child, marital conflict, lack of support from the family, past history of psychiatric illness, high parity, complications during pregnancy and low maternal education. Previous studies from low- and middle-income countries report similar risk factors.^58^^,^^67^

We found relatively higher pooled proportion of postpartum depression in mothers residing in urban than in rural areas. This may be due to factors such as overcrowding, inadequate housing, breakdown of traditional family structures leading to fragmented social support systems, increased work pressure, high cost of living and increased out-of-pocket expenditure on health care.^68^ Pooling of hospital-based studies found comparatively higher estimates of postpartum depression than studies in community settings. It is likely that mothers suffering from any illness during the postnatal period, including postnatal depression, will seek care at a health facility, compared to physically healthy mothers and babies who may not visit a facility at all. Moreover, being in a hospital environment provides an opportunity for the mother to express her concerns and problems to the health personnel, but when interviewed at her home she may not admit to having depressive symptoms, owing to the presence of other family members or neighbours and the social stigma attached to mental health conditions.

On subgroup analysis, we found a slightly higher proportion of postpartum depression in mothers who were aged > 25 years compared with those aged ≤ 25 years. Moreover, high maternal age emerged as a risk factor for depression in 4/28 studies which included this variable compared with 3/28 studies reporting low maternal age as a risk. Older mothers may suffer more from depression because they lack peer support or because they have more obstetric complications and multiple births or greater use of assisted reproductive technologies.^69^^–^^71^ On the other hand, it is possible that depression among older mothers is simply a biological phenomenon.

In our meta-analysis, geographical variation in the prevalence of postpartum depression was observed, with the highest prevalence in the southern regions. The observed differences in prevalence were not statistically significant on meta-regression and therefore more data are needed to document any significant geographical variations. The southern parts of the country have high literacy rates, which could lead to increased awareness about this health issue and therefore increased care-seeking.^72^ Moreover, the health system in southern India is more organized and there is comparatively better primary health-care provision than in other parts of the country and this could be a factor in greater care-seeking.^73^ South India also has a higher proportion of people living in urban slums compared with the northern parts of the country and greater rates of intimate partner violence.^74^^,^^75^

We found that the number of studies on postpartum depression has seen an upward trend in the last five years. There were 26 published studies between 2011‒2016, compared with six each in the periods 2000‒2005 and 2006‒2010. This reflects a recent interest of the medical research community towards this important issue.

There are a lack of data on perinatal mental health problems from low- and middle-income countries^76^ and this gap in the evidence hinders the process of establishing interventions to promote maternal psychosocial health. Gathering data on perinatal mental health issues will be essential in these countries, not only to gauge the magnitude of the problem, but also to inform policy-makers. Such evidence can stimulate governments to allocate resources for capacity-building in maternal mental health care, such as developing and implementing guidelines and protocols for screening and treatment, and setting targets for reducing the burden of postpartum depression. 
